# Exploring the Effects of Roadside Vegetation on the Urban Thermal Environment Using Street View Images

**DOI:** 10.3390/ijerph19031272

**Published:** 2022-01-24

**Authors:** Bin Li, Hanfa Xing, Duanguang Cao, Guang Yang, Huanxue Zhang

**Affiliations:** 1 College of Geography and Environment, Shandong Normal University, Jinan 250300, China; giserlibin@163.com (B.L.); cdguang@foxmail.com (D.C.); 2 Beidou Research Institute, Faculty of Engineering, South China Normal University, Foshan 528000, China; gyang@m.scnu.edu.cn; 3 School of Geography, South China Normal University, Guangzhou 510631, China

**Keywords:** spatial relationship, grass–shrub–tree, land surface temperature, geographically weighted regression model

## Abstract

Roadsides are important urban public spaces where residents are in direct contact with the thermal environment. Understanding the effects of different vegetation types on the roadside thermal environment has been an important aspect of recent urban research. Although previous studies have shown that the thermal environment is related to the type and configuration of vegetation, remote sensing-based technology is not applicable for extracting different vegetation types at the roadside scale. The rapid development and usage of street view data provide a way to solve this problem, as street view data have a unique pedestrian perspective. In this study, we explored the effects of different roadside vegetation types on land surface temperatures (LSTs) using street view images. First, the grasses–shrubs–trees (GST) ratios were extracted from 19,596 street view images using semantic segmentation technology, while LST and normalized difference vegetation index (NDVI) values were extracted from Landsat-8 images using the radiation transfer equation algorithm. Second, the effects of different vegetation types on roadside LSTs were explored based on geographically weighted regression (GWR), and the different performances of the analyses using remotely sensed images and street view images were discussed. The results indicate that GST vegetation has different cooling effects in different spaces, with a fitting value of 0.835 determined using GWR. Among these spaces, the areas with a significant cooling effect provided by grass are mainly located in the core commercial area of Futian District, which is densely populated by people and vehicles; the areas with a significant cooling effect provided by shrubs are mainly located in the industrial park in the south, which has the highest industrial heat emissions; the areas with a significant cooling effect provided by trees are mainly located in the core area of Futian, which is densely populated by roads and buildings. These are also the areas with the most severe heat island effect in Futian. This study expands our understanding of the relationship between roadside vegetation and the urban thermal environment, and has scientific significance for the planning and guiding of urban thermal environment regulation.

## 1. Introduction

There is evidence that the replacement of the natural environment by the urban fabric can lead to significant temperature increases as well as significant heat island effects [1]. In particular, in warm climate regions, the heat island effect significantly increases energy expenditures and carbon emissions [2], and these changes critically impact urban ecology and have major adverse effects on public health [3,4]. To address this issue, improved urban greenery is emerging as an effective mitigation measure and is becoming an important component of urban landscape design [5]. However, rapid urbanization has greatly altered original green space patterns, resulting in cities facing a dilemma involving limited land resources and insufficient urban green space [6]; thus, urban greening is gradually shifting toward a multilevel development pattern in which grasses, shrubs and trees are rationally distributed, and this hierarchy has become the focus of greening landscape design [7]. Therefore, it is necessary to perform accurate quantitative analyses of urban greening and its thermal effect, as these processes are important conditions for guaranteeing urban ecological security and sustainable development [8].

As roadsides are important public spaces in cities and important components of the urban fabric [9], many studies have constructed analytical models related to public health [10], air quality [11] and noise pollution [12] at the roadside scale. These environmental conditions play important roles in urban planning, as traffic heat emissions and carbon emissions are two of the main contributors to climate change [13]. In particular, roads in urban areas are often surrounded by buildings, thus forming a diversified thermal environment, and urban residents are directly exposed to the thermal environment in these regions. This complex thermal exposure often has adverse effects on human health [14]. Previous studies have confirmed that urban vegetation can achieve a cooling effect by expelling water during transpiration [15]. In addition, trees distributed along roadsides block direct sunlight, thus improving thermal comfort in their coverage areas and adjacent environments [16]. Therefore, it is necessary to study and fine-tune the spatial relationship between greenery and the thermal environment at the roadside scale.

In recent years, a large number of scholars have explored vegetation information and the interrelationships between vegetation and the thermal environment. These studies can be broadly divided into two categories. For instance, in one previous study [17], the authors chose the metropolitan area of Beijing, China, as the study area, and analyzed the macroscopic relationship between urban greening and the thermal environment. Another previous study [18] investigated the influence of the distance anisotropy of the urban heat environment on heat islands from the perspective of spatial patterns. Most of the studies that fall into this category involved qualitative work. Second, many scholars have focused on the construction of thermal environment simulation models for urban greening research, and have studied the effects of urban green landscapes from a relatively microscopic perspective. The presence of heat islands and the effect of green spaces on heat islands have been explored using the UrbClim boundary climate model [19]. One past study [20] investigated the influence of plant bioactivity on the thermal environment at the microscale through the use of a climate model. By analyzing small green spaces, another study [21] identified the types and structures of small green spaces that can effectively reduce temperatures in urban neighborhoods, while other researchers [22] considered the daily variation in the urban heat island effect and constructed a numerical simulation model to reasonably guide the arrangement of green areas and give full play to their cooling benefits. Most of these studies were micro-quantitative studies conducted in small areas. However, there is a lack of accurate quantitative information regarding different roadside vegetation types in areas in which a large number of roads cross the inner-city region due to scale specificity and data availability, and there is still a lack of both qualitative and quantitative analysis of roadside thermal environments.

With the development of big data, three-dimensional information, such as radar data and point cloud data, can be obtained. Recently, some scholars have introduced the three-dimensional greening of cities into roadside thermal environment research. For instance, one previous study [23] explored the role of greening on the vertical surface decks of buildings with the aim of improving the outdoor thermal environment. Another study [24] analyzed the optimal vertical greening patterns for indoor thermal comfort in regions with cold winters and hot summers. Other researchers used [25] terrestrial laser scanning technology to refine the vegetation canopy structure in cities and obtain the three-dimensional point cloud volume of leaves to analyze the cooling effects of different woodlands and their corresponding influential ranges. However, studies such as these are often difficult to conduct due to the time-consuming and laborious acquisition of 3D data. Furthermore, these studies failed to provide detailed information regarding the factors that control greening in urban thermal environments.

In summary, the effect of greening on the thermal environment has become a hot research topic, and the data used in traditional research on urban greening landscapes and the thermal environment have mainly been obtained from remote sensing images and field observations [26]. More thermal environment studies have taken a “top-down” observational approach based on remote sensing images. In past research, the characteristics of hierarchical urban greenery have been ignored, and new observation data are needed to assist these analyses. With the rapid development of sensing technology, we can access large amount of publicly available data. For example, street view images have been widely used in urban landscape research in recent years [27,28,29]. These data offer the possibility of capturing hierarchical greening information from street data and studying the thermal environmental effect of this information. One past study showed that asymmetric roadside greening patterns can significantly impact microclimates [30]. Some scholars have performed geometric urban canyon classifications based on street view images and deep multitask learning, and have noted that the classification results are closely related to the thermal environment [31]. These results all reflect the application prospects of street view images in thermal environment research. However, there is still a lack of research on the effects of different vegetation types on roadside thermal environments based on street view images.

Based on the above background information, this research attempts to use street view images to extract different types of vegetation on roadsides and analyze their layout and spatial relationships with the thermal environment. This study makes two main contributions. First, based on street view images, the grasses–shrubs–trees (GST) structures of the analyzed city streets are extracted, their spatial distribution patterns are analyzed, and the relationships of these patterns with the normalized difference vegetation index (NDVI) values obtained by traditional remote sensing methods are discussed. Second, in this roadside thermal environment research, the analysis is focused on the impact of GST greening type differences on the roadside thermal environment, and the results have significance for guiding urban vegetation planning to alleviate the heat island effect and carbon emissions.

The rest of this article is organized as follows. Section 2 introduces the research methods of this study. The third section introduces the remote sensing retrieval results, the quantitative greening pattern results derived from street view pictures, and the analysis results of the spatial relationship between the greening derived from the street view images and the thermal environment data derived based on the geographically weighted regression (GWR) model. The fourth section discusses the difference between the greening information extracted from the street view images and the NDVI values used in urban thermal environment research and summarizes the shortcomings of this research study. The fifth section contains the conclusion of this research.

## 2. Materials and Methods

### 2.1. Research Framework

**Figure 1:**
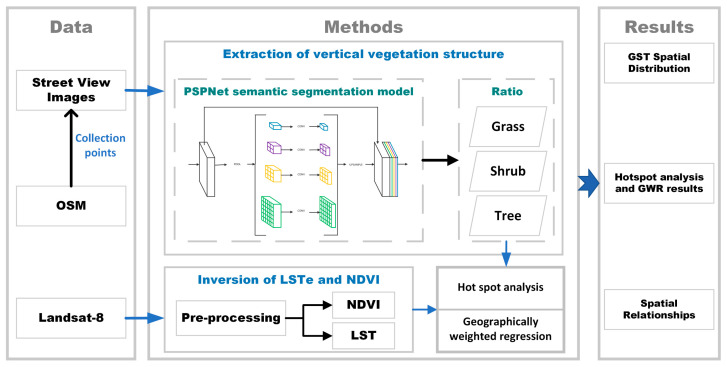
A sketch map of the research methodology.

The framework proposed in this paper consists of three main parts. The overall framework is shown in Figure 1. OpenStreetMap (OSM) road network data, Baidu map street view images and Landsat-8 remote sensing image data were obtained. In the second part, GST, NDVI and land surface temperature (LST) data were extracted from street view images (GST) and Landsat-8 images (NDVI and LSTs). Based on a hot spot analysis, the cold and hot spots of different roadside vegetation types were analyzed, and the spatial relationship between GST and LST was analyzed using GWR. The third section contains the results.

### 2.2. Extraction of GST from Street View Images

#### 2.2.1. Semantic Segmentation Network Model

The automatic extraction of image elements using semantic segmentation models has been proven to be extremely effective, and this method has been widely used to process street view images [32]. Therefore, we introduce the pyramid scene parsing network (PSPNet), an improved semantic segmentation network based on a full convolutional network (FCN). The core idea of this network is that when judging small local targets, the false recognition probability can be relatively reduced if more global information is introduced in the segmentation layer [33,34]. The main structure can be roughly divided into 3 parts: the ResNet convolution module for feature extraction, the pyramid pooling module and the final FCN output module. To reduce the amount of work required in the parameter computation process, a 1 × 1 convolution kernel is used to downscale the input data and then perform the convolution operation, and a convolution kernel of the same size is used to restore the data dimensionality of the output to that at the time of input. After the street view images are convolutionally pooled, the extracted feature maps are convolved in the pyramid pooling layer, which has four levels, and the sizes of the convolution kernels are 1 × 1, 2 × 2, 3 × 3 and 6 × 6. The size before unpooling is obtained via bilinear interpolation, this size is cascaded and integrated with the output, and the pixels are finally classified using the classifier to complete the street view image segmentation. In this study, based on the utilized network, different types of vegetation elements are extracted from street view images. Then, the numbers of pixels containing the different elements are calculated. The formula used to extract the elements is as follows:

(1) $$E_{i,\mathrm{object}}=\overset{4}{\underset{o=1}{\sum }}{\mathrm{Pixel}}_{o,\mathrm{object}}$$

where ${\;E}_{i,\mathrm{object}}$ refers to the number of pixels of the elements in the street view image at the ith street view collection point and o is the direction of the street view image at the street view collection point. This study selected five pictures oriented in five directions: front, back, left, right and top. In the above equation, ${\mathrm{Pixel}}_{o,\mathrm{object}}$ represents the number of pixels of the element object in the picture oriented in the direction of the collection point o. In this study, the actual number of segmented objects includes 15 elements, such as buildings, roads and sky. The vegetation types are subdivided into grasses, shrubs and trees, leading to the GST segmentation results. For the model validation, we use the intersection over union (IoU) method to evaluate the model performance. Specifically, IoU is the number of overlapping pixels between the predicted value and the true value divided by the union of the predicted value and the true value. The IoU method is a common method for judging the quality of a model in the image segmentation field [35].

#### 2.2.2. Construction of Different Vegetation Indicators

In the research field of landscape ecology, the landscape index has been introduced [36]. By analogy with the semantically segmented category elements, the emphasis is placed on grasses, shrubs and trees that reflect the greening of the roadside, and their abundances on the roadside are then measured. The measurement formula [37] and its description are shown in Table 1.

### 2.3. Extraction of NDVI and LST from Landsat-8 Image

According to the radiative transfer equation algorithm, the thermal infrared bands of Landsat-8 remote sensing images are used to retrieve the surface temperature data of the study area, and the thermal infrared band is resampled with a spatial resolution of 30 m to facilitate subsequent surface temperature calculations. This algorithm is a conventional surface inversion algorithm based on the atmospheric radiation transmission model. It can subtract the atmospheric influence deviation value from the total thermal radiation value observed by satellite sensors to obtain the surface thermal radiation intensity and convert it to the corresponding LSTs. The formula of this algorithm is shown below:

(2) $$T_{s}=\frac{K_{2}}{\ln{\left(\frac{K_{1}}{B\left(T_{s}\right)}+1\right)}^{\prime }}$$

(3) $$B\left(T_{s}\right)=\frac{\left[L_{\lambda }-L_{↑}-\tau \left(1-\varepsilon \right)L_{↓}\right]}{\tau \varepsilon }$$

where $T_{s}$ is the true LST, ε is the surface-specific emissivity, τ is the atmospheric transmittance in the thermal infrared band, $L_{\lambda }$ is the image radiometric calibration, $B\left(T_{s}\right)$ is the blackbody emissivity, $L_{↑}$ is the atmospheric upward radiant intensity, $L_{↓}$ is the atmospheric downward radiant intensity and $K_{1}$ and $K_{2}$ are coefficients. In the Landsat-8 thermal infrared band, K_1_ = 774.885 W·m^−2^·sr^−1^·μm^−1^ and K_2_ = 1321.079 K. From the National Aeronautics and Space Administration (NASA) website (http://atmcorr.gsfc.nasa.gov, accessed on 1 September 2021), we can obtain the following values for τ, $L_{↑}$ and $L_{↓}$, which are 0.72, 2.18 and 3.46, respectively.

To derive the NDVI, the difference between the near-infrared band, which is strongly reflected by vegetation, and the red light band, which is absorbed by vegetation, is used to quantify the vegetation coverage. This index can eliminate most of the variation in irradiance related to the effects of instrument calibration, topography, shading, etc. NDVI enhances the response to vegetation and is the most widely used vegetation index available today. NDVI is calculated as follows:

(4) $$\mathrm{NDVI}=\frac{\left(\mathrm{NIR}-\mathrm{Red}\right)}{\left(\mathrm{NIR}+\mathrm{Red}\right)}$$

where NIR represents the near-infrared band, Red represents the red band and the final NDVI result is between −1 and 1. Generally, the larger the NDVI value is, the higher the vegetation cover is.

### 2.4. Statistical Method of Determining Hot/Cold Spots

Ignoring spatial variations may lead to incorrect conclusions in the analysis process. Therefore, we used a hot spot analysis to characterize the heterogeneity of the LST and GST values within cities. In this hot spot analysis, the Getis-Ord Gi* statistic is used to identify statistically significant hot and cold spots [38]. This method calculates the Getis-Ord Gi* statistic for each element in the dataset, and the resulting z score and *p* value indicate how the high-value and low-value elements are clustered in space. If the z score of an element is high and the *p* value is small, these results indicate the existence of a high-value spatial cluster. If the z score is low and negative and the *p* value is small, then a low-value spatial cluster exists. The higher (or lower) the z score is, the greater the degree of clustering is. If the z score is close to zero, there is no significant spatial clustering. In existing analytical research, this tool has been widely used to identify spatial clusters with statistically significant high values (hot spots) and low values (cold spots).

At the same time, a boxplot is generated in this study to reveal the spatial differences in green space information between cold spots and hot spots on roadside LSTs. Four indicators, the minimum, maximum, mean and standard deviation values, were selected to measure the differences between cold and hot spots.

### 2.5. Geographically Weighted Regression Model

GWR combines spatial correlation and linear regression methods to improve traditional models [39]. Compared with traditional models, such as the ordinary least squares (OLS) model, GWR has certain advantages because it supports both independent variables and dependent variables. The parameter estimations reflecting the local changes in the correlations among the variables can affect the relationships among the variables due to changes in their geographical position, and these changes can reflect the local characteristics that are ignored. The model structure is as follows [40]:

(5) $$y_{i}=\overset{j=n}{\underset{j=0}{\sum }}\beta_{\mathrm{ij}}{\times x}_{\mathrm{ij}}+\varepsilon_{i}$$

where $y_{i}$ is the dependent variable at the ith location, $x_{\mathrm{ij}}$ is the independent variable, $\beta_{\mathrm{ij}}$ is the coefficient of the jth independent variable at the ith location and $\varepsilon_{i}$ is the error term. Based on the above information, this paper uses the GST ratio indicator as the independent variable and the LST as the dependent variable. In addition, to evaluate the effect of implementing GWR, in addition to testing the collinearity of parameters, we introduce the Akaike Information Criterion (AIC) to measure the goodness of fit of the statistical model, which can weigh the complexity of the estimated model and the fit of the model to the data [41]. Finally, the OLS model and GWR model were evaluated based on AIC and adjusted R^2^ values.

## 3. Results

### 3.1. Study Area and Data

**Figure 2:**
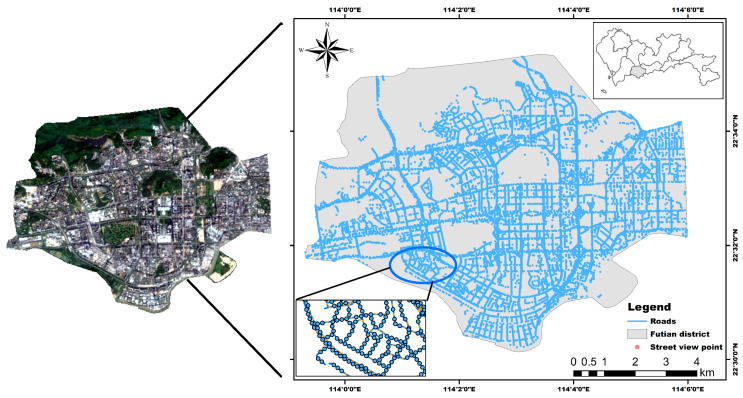
Visualization of the study area and basic data.

This article takes Futian District, located in Shenzhen, Guangdong Province, as the study area (Figure 2); this district has a total area of 78.66 km^2^ and a resident population of 1.517 million people. Futian District is the political, economic and cultural center of Shenzhen and has a subtropical monsoon climate. Due to sufficient light and heat throughout the year and the hot and humid local climate, Futian is among the cities with the most serious heat island effects.

According to the location of the study area, we used the Landsat-8 image taken at approximately 10:52 a.m. Beijing time on 2 November 2019 to retrieve the LSTs. This Landsat-8 image has a spatial resolution of 30 m and consists of 11 spectral bands, including nine multispectral bands, one panchromatic band, and two thermal infrared bands. Landsat-8 is currently one of the most commonly used data sources for retrieving LSTs. Taking into account the data availability, we compared the historical weather data of Guangzhou and found that the highest temperature recorded on this day in Guangzhou was 30 °C, the weather was fine, and a northeast wind was present at two levels. This information suggests that the Landsat-8 image meets the requirements for this application. Moreover, the latest road data for the area were acquired from OSM, in which street images were captured at sampling points located every thirty meters along the road, and a total of 19,596 street images were obtained after poor-quality images (such as images taken inside tunnels) were removed.

### 3.2. Vegetation Information and LST Extraction Results

#### 3.2.1. Extraction Results of Different Vegetation Types from Street View Images

**Figure 3:**
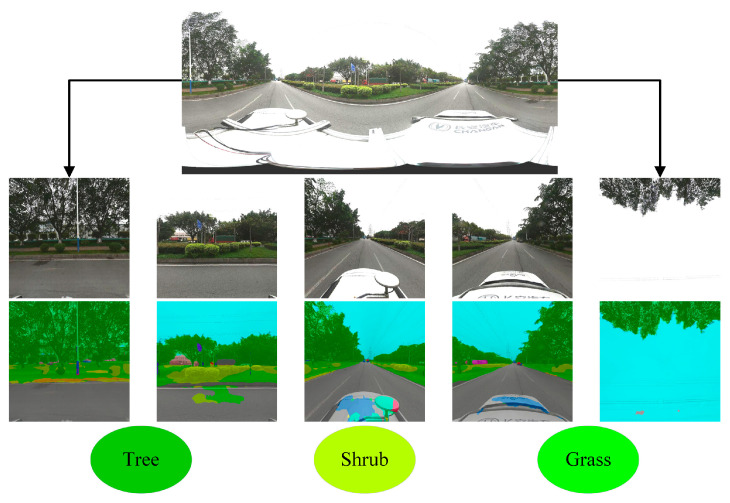
Extraction results of vegetation types from street view images.

In this paper, the PSPNet semantic segmentation network was used to extract elements, including grasses, shrubs, trees and other elements, and this network achieved excellent performance (Figure 3). In particular, the model predicted results with an accuracy of up to 77.23% in Cityscapes (a large-scale dataset containing high-quality pixel-level annotations of 5000 images of 50 cities taken in different seasons) [42]. In the tenfold cross-validation process, the mean IoU value derived for the independent test set was 83.8%, and the mean IoU values derived for trees, shrubs and grasses reached 89.6%, 73.3% and 84.1%, respectively. These high-accuracy GST segmentation results confirmed the feasibility of using street view images to extract different vegetation types from roadsides, thus providing a database for the subsequent analysis.

#### 3.2.2. LST and NDVI Extraction Results

**Figure 4:**
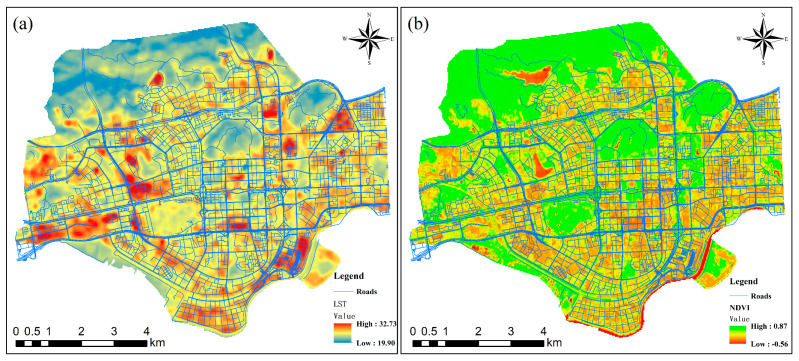
Mapped retrieval results of Landsat-8 remote sensing images: (**a**) LST retrieval results and (**b**) NDVI retrieval results.

Figure 4 shows the spatial distribution of the LST and NDVI retrieval results in the entire study area. The LSTs range from 19.90 °C to 32.73 °C, and the temperature difference reaches 12.83 °C. Among the results, the area with the lowest LST is located on the surface of a water body, and the area with the highest LST is located in a densely built area. The area with the lowest NDVI value is located on the surface of a water body, and the area with the highest NDVI value is located in a woodland. The results show that the urban thermal environment has significant spatial clustering characteristics, especially the surface thermal environment, which is extremely complex. At the same time, the clustering characteristics of high-value urban NDVI areas are also prominent, reflecting the good overall vegetation in Futian District and revealing a north–south pattern. An increasing trend was observed, but the vegetation coverage rate inside the city was relatively low, and more vegetation was distributed on the sides of roads. In particular, due to the occlusion by the large tree canopy and man-made facilities such as buildings, it is difficult to capture the information of low vegetation, such as shrubs and grasslands, at the bottom, which means that the inversion results for the remote sensing images have errors, and cannot accurately measure road greening or the different cooling effects related to different types of vegetation.

### 3.3. Hot Spot Analysis and Statistical Analysis Results

**Figure 5:**
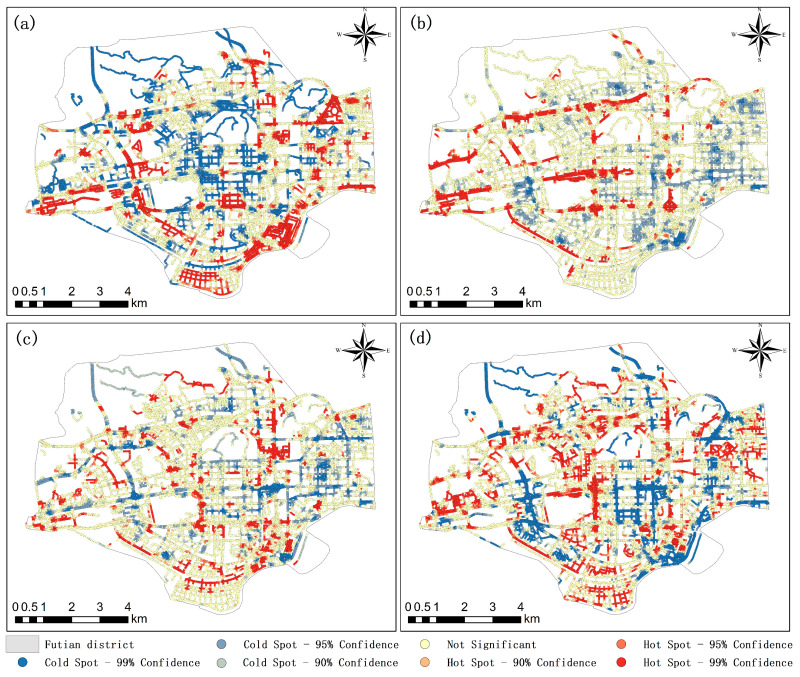
Hot spot analysis results of the correlation factors: (**a**) LST hot spot analysis results, (**b**) grass ratio results, (**c**) shrub ratio results and (**d**) tree ratio results.

The hot spot analysis results show that the GST distribution in Futian District is significantly spatially autocorrelated. Figure 5 shows the LST and GST hot spot analysis results. Figure 5a reveals that the areas with higher LSTs are mainly concentrated in the southern industrial zone of Futian District, the western region close to the Futian Bus Station, the northeastern Futian Stadium and the southeastern customs region. In the interior of the city, there is no large area containing high LSTs. Hot spot clusters and prominent hot spots are distributed in small blocks. At the same time, the figure also reveals the complex thermal environment in Futian District. Figure 5b–d depicts the spatial clustering of hot/cold spots containing different vegetation types. The hot spots depicted by the red areas in Figure 5 can be regarded as high-coverage areas of the corresponding vegetation type, while the cold spots depicted by the blue areas in the figure can be regarded as areas containing sparse coverage of the corresponding vegetation types. Focusing on the distributions of different vegetation types, the difference between GST and NDVI can be clearly observed. Specifically, Figure 5b shows that grasses are mainly distributed on several main roads that pass through the inner city, while there is very little grass on either side of the low-grade roads in the city blocks. This distribution is due to the main trunk roads designed during urban planning. Regarding road greening measures, in the eastern part of Futian District, grass is not the main vegetation type and forms a partial, intermittent cold spot. The NDVI in this area is almost entirely supported by other vegetation types. Figure 5c shows that shrubs do not form any large continuous distribution, but are still the main vegetation type throughout the entire study area; shrubs are mainly distributed along secondary roads and outer ring roads within the city. In places where shrubs are densely distributed, high-density grass or tree coverage is rarely observed at the same time. This shows that shrubs and other vegetation types are rarely present together. The existence of a large grassland distribution would put pressure on urban planning. Figure 5d shows that trees are still the most widely distributed vegetation type, and their distribution is most similar to the NDVI search results. At the same time, the hot spots shown in the figure often contain shrubs and grasses, suggesting that Futian District is in the planning stage of urban greening and that attention should be given to the ratio of different vegetation types. In general, the NDVI results have the highest similarity with tree coverage, followed by shrub coverage and grass coverage. The cold and hot spots of these three types of vegetation overlap and differ, revealing complex greening conditions in the study area.

**Figure 6:**
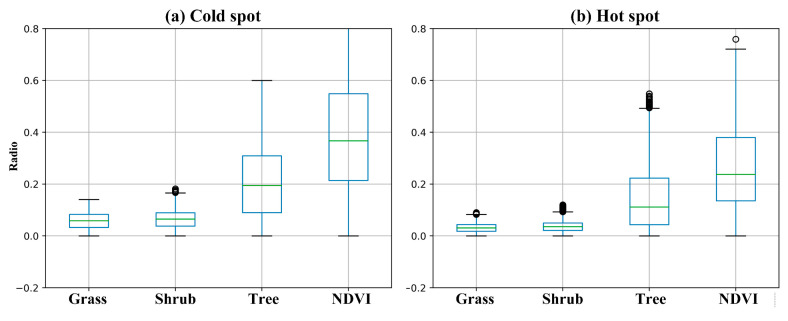
Boxplots depicting the statistical information of the GST and NDVI results at (**a**) cold and (**b**) hot spots.

To explore the significant differences between the GST and NDVI results in association with the LST cold and hot spots, we calculated statistics for the cold and hot spots (Figure 6). In general, the greenery in Futian District has a relatively high level, indicating that the vegetation coverage is high in most areas. In particular, in cold spot areas, the maximum and average GST and NDVI values were significantly higher than those in hot spot areas, and both the tree ratio and NDVI values in cold and hot spot areas showed a large range, while both the grass and shrub ratios showed smaller ranges. These results show that only a few roadside tree coverage and other vegetation coverage rates are high, thus explaining the spatial heterogeneity of the tree distribution. In contrast, the grass and shrub distributions are relatively balanced, and there is no absolutely prominent regional distribution of either of these vegetation types.

### 3.4. Spatial Relationship Analysis Based on GWR

To better consider the spatial heterogeneity of the impacts of GST on LSTs, a GWR model was used to explore the spatial relationships between different vegetation types and the urban thermal environment. First, to prevent the occurrence of bias in the estimation results due to GST interactions, a covariance test was performed on the indicators (Table 2). The results of the covariance test showed that the variance expansion factor value of each indicator was less than 5, and the corresponding conditional index values were also less than 5; thus, there was no covariance among the GST indicators, and the GWR model was suitable for analyzing this type of index.

**Figure 7:**
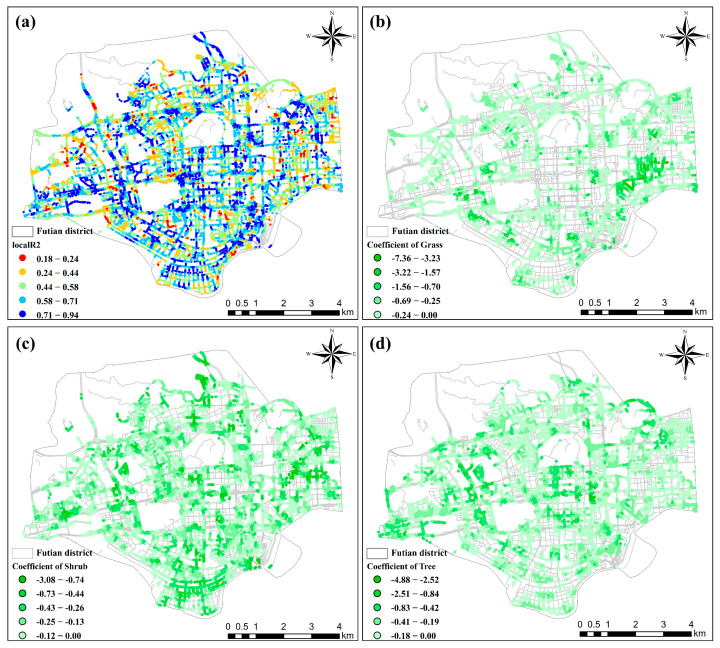
GWR model results: (**a**) local R^2^ values of GWR, (**b**) grass ratio coefficients, (**c**) shrub ratio coefficients and (**d**) tree ratio coefficients.

We further compared the performances of the two models, OLS and GWR. Table 3 shows that the GWR model had significantly lower AIC values and higher adjusted R^2^ values compared to the OLS model. In the GWR model results, as shown in Figure 7a, the regression results performed well in a large part of the study area, the R^2^ values of most areas were high and each vegetation factor had specific characteristics in different areas. The regression coefficients were mostly negative, reflecting the same results as those obtained in previous studies regarding the important role of greening in adjusting the urban thermal environment.

As shown in Figure 7a, the regions with low local R^2^ values were more scattered, while regions with high local R^2^ values were more concentrated and more widely distributed. The dense area in the middle of the city maintained its high fitting results, and the range of R^2^ values in this region was from 0.18 to 0.93. Among these areas, the sampling points with R^2^ values greater than 0.6 accounted for 55% of the total area. This shows that the vegetation information extracted from the street view images can be used to detect thermal environmental conditions; specifically, in urban areas with high artificial surface densities, the best fitting effect can be achieved, thus confirming the usability of the GWR model. Figure 7b shows the areas where the grassland percentage regression coefficients were less than 0. The grassland in these areas has a good cooling effect, and the minimum regression coefficient reaches −7.36. However, by comparing the distribution hotspots of grassland, it can be seen that the areas with a better cooling effect do not have higher grassland coverage. The degree of overlap between the two is quantified as a percentage and is only 25.3%. At the same time, the cooling effect of grass is less than that of other types of vegetation near the main roads in the city center. In contrast, the grasses have better cooling effects in the eastern part of Futian District, that is, in the commercial and residential centers of the district, and there is no continuous grass distribution hot spot in this area. Figure 7c shows the area where the regression coefficient of the shrub ratio is less than 0; the smallest regression coefficient is −3.07. The overall cooling effect of shrubs is not particularly significant, but areas corresponding to shrub cooling effects are more widely distributed than those of grasses and trees. Sampling points with negative regression coefficients cover 73% of the total points, and these points are especially located in the commercial area in the center of the city and the industrial area in the southern part of the city where the shrub distribution hot spots overlap more with areas corresponding to excellent cooling effects, with an overlap of 47.9%, thus confirming the advantages of mixed greenery. Although the distribution of shrubs was not concentrated, shrubs played a vital role in the local cooling of the entire study area. Figure 7d shows the area in which the tree percentage regression coefficients were less than 0; the minimum regression coefficient reached −4.87. It is worth noting that the sampling points with negative regression coefficients account for 70% of all sampling points, and there is a tendency for more clustering in areas with good cooling effects. The overlap between these areas and the hot spots of tree distribution reaches 43.4%, which indicates that in most areas, trees can have a good cooling effects, and better vegetation planning is expected to connect the areas with cooling effects into larger patches. Compared with the other two types of vegetation, the areas where trees had a cooling effect were best fitted to the tree distribution hot spots. Among these areas, the areas with the best cooling effect were located in the central area of Futian District, and the main roads in this area crisscross, with dense buildings and dense traffic. The distribution of trees in this region provides the best cooling effect.

For different types of vegetation, the hot spot distributions overlap and differ from areas with cooling effects, thus indicating that the existing vegetation greening has played a part in mitigating the heat island effect, but that there is still a lack of the planning required to give full play to the cooling effect of vegetation. After estimating various factors in a specific area, a specific vegetation type should be selected, and the mixed greening model should also become the focus of urban greening in the future.

## 4. Discussion

The method proposed in this paper provides a new process for studying different vegetation types and urban thermal environments. In the proposed method, street view images are used to extract different types of vegetation and identify and analyze their cooling performances in an urban thermal environment. In this paper, we both enhance the existing research regarding the distribution structure of urban vegetation types and analyze the spatial variabilities of different vegetation types that affect the urban thermal environment. The research results can expand the scientific understanding of the influence of urban vegetation structures on LSTs and provide references with which urban planners can mitigate the urban heat island effect by optimizing the spatial distribution of urban vegetation.

### 4.1. Performance Comparison between GST and NDVI

The results show that the GST greening index is significantly negatively correlated with the LST; this has been widely accepted by most researchers [43,44]. However, studies on the relationships between different vegetation types and the urban thermal environment are still scarce. In previous studies, to fully consider the vertical tree morphology structure, a forest canopy model was developed to more accurately simulate the thermal impacts of afforestation on urban meteorological behavior [45]. However, the accuracy of this model is biased due to the difficulty of collecting real measured parameter values. Some scholars have also studied the relationship between LSTs and urban vegetation configurations at multiple scales using remote sensing imagery [46]. However, this is still a top-down observation approach that ignores the vertical structure of urban greenery and does not refine the quantification process of the thermal impacts of different vegetation types. Therefore, we introduced the use of street view imagery to extract street vegetation information; however, due to the data themselves, uncertainty is still present when these images are used to study urban thermal environments, and it is necessary to compare the derived results with remotely sensed images.

**Figure 8:**
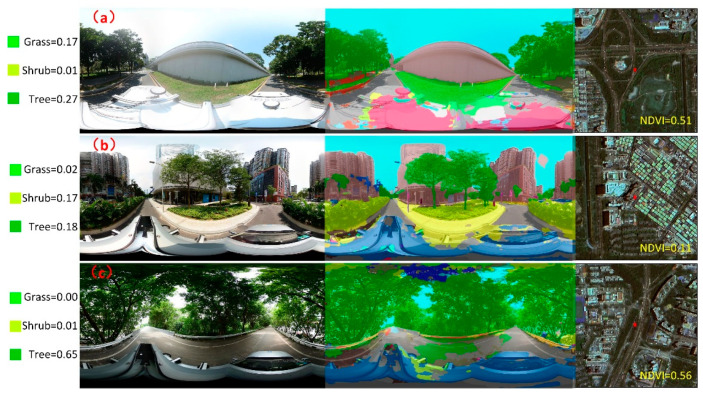
Comparison of street view images and remote sensing images (the locations at which the street view images were taken are labeled in the remote sensing images by red markers). (**a**) Grass dominated; (**b**) Shrub dominated; (**c**) Tree dominated.

When describing the vegetation in the same area using street view and remote sensing imagery, there is a clear difference in the derived vegetation information (Figure 8). Figure 8 shows several examples of significant differences between street view images and remote sensing images within the study area. In Figure 8a, continuous grass coverage and surrounding distributed trees can be observed in the street view images; due to the flat terrain and suburban location, the same large-scale vegetation distribution is observed in the remote sensing image with an NDVI value of 0.51. Similar patterns are exhibited in the analysis results based on the street view images. In Figure 8b, the layout of trees and shrubs can be observed in the study area, and the greenery is still sufficiently detailed that trees and shrubs can be effectively classified. In the remote sensing image in Figure 8, because the area shown is located in the center of a neighborhood and is surrounded by tall buildings, the NDVI value of the top-down remote sensing view is only 0.11, and this value does not truly reflect the greenery of the area due to obscuration and other reasons, for example, the quality of remote sensing images. This is another important aspect of the discrepancies between NDVI retrieval results and shrub and grass recognition results. Figure 8c shows the distribution area of large-scale continuous trees, the main vegetation type in the open spaces on both sides of the main road; the results derived from the street view image segmentation process achieved similar results to those reflected by the NDVI values. From this perspective, street view images can compensate for the deficiencies present in the top-down observational perspective of remote sensing images to a certain extent, and they have their own advantages in the subdivision of vegetation types and vegetation structure layout analyses.

### 4.2. Additional Factors Influencing the Urban Thermal Environment

This paper identifies that street view images have great potential for studying the roadside thermal environment. Vegetation has been shown to be an extremely important source of urban cooling effects [47], but LSTs can have high heterogeneity due to factors other than vegetation. Other relevant factors also influence the urban thermal environment due to the highly complex nature of the urban landscape. Similar to the building density [48], the distribution of water bodies [49], road orientation [50], etc., high-density areas of artificial surfaces, such as buildings and roads, change the characteristics of the urban subsurface and often lead to the emergence of urban heat island core areas [51]. Furthermore, the distribution of large areas of water and vegetation can present a significant cooling effect due to these coverage types having high specific heat capacities and low reflectivities. The cooling effect of vegetation at the roadside scale considered in the present study may be simultaneously influenced by nearby urban areas with higher temperatures. For example, in Futian District, the areas where trees are widely distributed are often areas with greater development intensity and more anthropogenic heat release. The distribution of the cooling effect of arbors is not the most widespread, which has been confirmed by the results of the GWR model. Urban landscapes moderate the relationship between LST and vegetation at small scales within cities, resulting in low- and high-temperature patches, but they are often neglected in research on the cooling effect of vegetation. The large differences between these patches, especially on the roadside scale, have suggested that an unbalanced distribution of these patches leads to a significant separation of the thermal environment within a city [52], and thus provides us with the opportunity to study the roadside thermal environment.

### 4.3. The Scaling Problem of the Cooling Effect

Here, we discuss the scale at which street view images have the best goodness of fit when studying roadside thermal environments. Since street view images are collected as points on roads, and the heat propagation intensity and direction are uncertain, the optimal scale for determining the cooling effect of roadside vegetation should be considered, which is crucial for practical urban vegetation landscape planning. In most cases, the closer the area is to the vegetation, the better the cooling effect is. An increase in the influence radius will inevitably lead to a decrease in the vegetation cooling effect. Meanwhile, considering that our main focus is on the roadside and that the spatial resolution of Landsat-8 remote sensing imagery is 30 m, the distance interval between street collection points was set to 30 m, and the buffer zone was 30 m. In this study, the final GWR model fit reached 0.835. Despite this good fit, the magnitude of the cooling effect still needs to be explained. With this in mind, a more objective result was obtained in this study using a multilevel buffer analysis.

**Figure 9:**
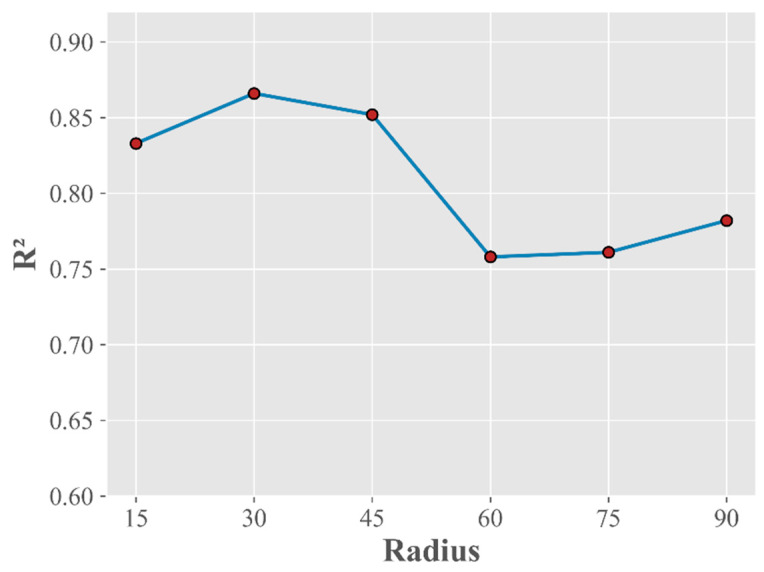
GWR model fitting results obtained at different analysis scales, the X axis represents the length of the radius, and the Y axis represents the model fit R².

Taking into account the widths and vegetation plans of conventional urban roads, we designed six sets of comparative experiments with buffer radii ranging from 15 to 90 m, centered on the streetscape acquisition point, to determine the best-fitting scale of roadside vegetation and LSTs. We calculated the average temperatures of all image elements within these buffers and performed model fitting. The results of the analysis are shown in Figure 9; the optimal scale for the fitting effect was 30 m, followed by 45 m. As the radius increased, the fitted R^2^ values significantly decreased. Taking into account the specific needs of actual vegetation planners, it is also necessary to conduct a more in-depth discussion on the scaling of the cooling effects. In particular, hierarchical vegetation planning plays an important role in guiding urban planning. Existing studies have shown that the cooling of vegetation is affected by the type of vegetation and its influence range. As the influence range becomes larger, the vegetation has a smaller cooling effect [53]. The ventilation environment means that the thermal environment of the road is more affected by the scale [54]. Buffer analysis showed that 30 m was a good fitting scale for the cooling effect of roadside vegetation. In the actual urban planning process, more consideration should be given to the impact of roadside vegetation only on the thermal environment of the road. At the same time, the research method based on street view images has good transferability, and the corresponding vegetation type in the study area has an excellent cooling effect. Different types of vegetation can be deployed in a targeted manner by considering various factors, which are also two important aspects of future research.

### 4.4. Limitations and Future Avenues

Research has shown that street view images have great potential for use in research on urban thermal environments. However, the current study still has some limitations. First, the street view data are distributed as point data. Considering that heat decays with distance, there are still some shortcomings when considering the anisotropy of heat [55]. Second, the LSTs retrieved herein contained some errors attributed to various reasons, such as sensor uncertainty [56], but these values were still generally usable and could explain the spatial layout of and spatial relationships among vegetation types. Therefore, in future research, more attention should be given to determining how to better process streetscape data, how to improve the LST retrieval accuracy and how to combine street view images with remote sensing images to obtain better fitting results.

## 5. Conclusions

Street view images contain rich information regarding geographic locations and the surrounding landscape, and the ability of these images to capture the greenery information of urban roads in the horizontal view is especially noteworthy. This study confirms that street view images can complement the “top-down” shortcomings of remote sensing images in thermal environment research. Street view images have great potential in urban thermal environment research, but have not yet been effectively analyzed or applied. In this paper, the spatial layouts of different vegetation types and their mediation effects on the urban thermal environment are analyzed by applying street view images to extract different types of vegetation at the roadside scale. It is concluded that (1) areas experiencing significant cooling effects due to the presence of grasses are mostly located in commercial areas. In addition, areas with excellent cooling effects differ from the grass distribution hot spots, indicating that the planning of multitype vegetation ratios in urban greening planning is lacking. (2) Areas with cooling effects due to the presence of shrubs are most widely distributed, and the cooling effect of shrubs is best in the industrial areas of the studied city. The distribution hot spots heavily overlap with the areas experiencing cooling, confirming the advantages of mixed greening with trees and shrubs. (3) Areas with the best cooling effect resulting from the presence of trees are usually located in urban areas with dense buildings and roads. These areas have the highest similarity with the NDVI retrieval results. The spatial heterogeneity of different vegetation types regulating the thermal environment was also demonstrated. In conclusion, street view images are more optimal for quantifying road vegetation information from the pedestrian perspective than remote sensing images. In actual urban planning and management projects, the type, structure and spatial layout of urban vegetation may need to be considered if appropriate decisions for more effective regulation in the context of urban heat island mitigation are to be made. The main findings of this paper provide insight into the cooling effects of different vegetation types at the street scale within cities, and thus provide useful insights for future research on the thermal environments of urban roads and site-specific vegetation layout planning.

## Figures and Tables

**Table 1:** Description of the indicators used to quantify the different vegetation types.

Name	Definition
Grass	$\mathrm{Grass}=\frac{E_{\mathrm{grass}}}{\mathrm{All}\;\mathrm{Pixels}}$, where $E_{\mathrm{grass}}$ represents the number of pixels occupied by the grass element in the street view image and All Pixels represents the total number of pixels
Shrub	$\mathrm{Plant}=\frac{E_{\mathrm{shrub}}}{\mathrm{All}\;\mathrm{Pixels}}$; similar to the grass equation, ${\;E}_{\mathrm{shrub}}$ represents the ratio of the shrub area to the total area of the street view image
Tree	$\mathrm{Tree}=\frac{E_{\mathrm{tree}}}{\mathrm{All}\;\mathrm{Pixels}}$; similar to the grass equation, $E_{\mathrm{tree}}$ represents the ratio of the tree area to the total area of the street view image

**Table 2:** Collinearity inspection of the influential factors.

Variables	VIF	Tolerance	Condition Index
Grass	1.044	0.958	3.731
Shrub	1.085	0.922	2.498
Tree	1.122	0.9891	2.028

**Table 3:** Comparison between GWR and OLS results.

	OLS-AIC	OLS-Adjust R^2^	GWR-AICc	GWR-Adjust R^2^
Model	31,376.851	0.388	14,286.048	0.865

## Data Availability

Not applicable.
